# Making PBPK models more reproducible in practice

**DOI:** 10.1093/bib/bbae569

**Published:** 2024-11-04

**Authors:** Elena Domínguez-Romero, Stanislav Mazurenko, Martin Scheringer, Vítor A P Martins dos Santos, Chris T Evelo, Mihail Anton, John M Hancock, Anže Županič, Maria Suarez-Diez

**Affiliations:** Department of Bioinformatics—BiGCaT, School of Nutrition and Translational Research in Metabolism, Faculty of Health, Medicine and Life Sciences, Maastricht University, P.O. Box 616, 6200 MD, Maastricht, The Netherlands; RECETOX, Faculty of Science, Masaryk University, Kotlarska 2, 611 37, Brno, Czech Republic; Loschmidt Laboratories, Department of Experimental Biology and RECETOX, Faculty of Science, Masaryk University, Kamenice 5, 625 00, Brno, Czech Republic; International Clinical Research Center, St. Anne’s University Hospital Brno, Pekařská 53, 656 91, Brno, Czech Republic; RECETOX, Faculty of Science, Masaryk University, Kotlarska 2, 611 37, Brno, Czech Republic; Laboratory of Bioprocess Engineering, Wageningen University and Research, Droevendaalsesteeg 1, 6708 PB, Wageningen, The Netherlands; Department of Bioinformatics—BiGCaT, School of Nutrition and Translational Research in Metabolism, Faculty of Health, Medicine and Life Sciences, Maastricht University, P.O. Box 616, 6200 MD, Maastricht, The Netherlands; ELIXIR, Wellcome Genome Campus, Hinxton, Cambridgeshire CB10 1SD, United Kingdom; Department of Life Sciences, National Bioinformatics Infrastructure Sweden (NBIS), SciLifeLab, Chalmers University of Technology, Kemigården 1, Gothenburg SE-412 96, Sweden; Institute of Biochemistry and Molecular Genetics, Faculty of Medicine, University of Ljubljana, Zaloška 4, 1000 Ljubljana, Slovenia; Department of Biotechnology and Systems Biology, National Institute of Biology, Večna pot 121, 1000 Ljubljana, Slovenia; Laboratory of Systems and Synthetic Biology (SSB), Wageningen University and Research, Agrotechnology and Food Sciences, Stippeneng 4, 6708 WE Wageningen, The Netherlands

**Keywords:** systems biology, pharmacokinetics, model code, reproducibility, SBML, MATLAB

## Abstract

Systems biology aims to understand living organisms through mathematically modeling their behaviors at different organizational levels, ranging from molecules to populations. Modeling involves several steps, from determining the model purpose to developing the mathematical model, implementing it computationally, simulating the model’s behavior, evaluating, and refining the model. Importantly, model simulation results must be reproducible, ensuring that other researchers can obtain the same results after writing the code *de novo* and/or using different software tools. Guidelines to increase model reproducibility have been published. However, reproducibility remains a major challenge in this field. In this paper, we tackle this challenge for physiologically-based pharmacokinetic (PBPK) models, which represent the pharmacokinetics of chemicals following exposure in humans or animals. We summarize recommendations for PBPK model reporting that should apply during model development and implementation, in order to ensure model reproducibility and comprehensibility. We make a proposal aiming to harmonize abbreviations used in PBPK models. To illustrate these recommendations, we present an original and reproducible PBPK model code in MATLAB, alongside an example of MATLAB code converted to Systems Biology Markup Language format using MOCCASIN. As directions for future improvement, more tools to convert computational PBPK models from different software platforms into standard formats would increase the interoperability of these models. The application of other systems biology standards to PBPK models is encouraged. This work is the result of an interdisciplinary collaboration involving the ELIXIR systems biology community. More interdisciplinary collaborations like this would facilitate further harmonization and application of good modeling practices in different systems biology fields.

## Introduction

Systems biology is a scientific field that aims to understand living organisms as whole systems, by modeling them at different organizational levels [1, 2]. Key challenges in systems biology include the resistance to mathematical modeling in biological sciences and the need to improve the comprehensiveness, ‘reproducibility’, and ‘interoperability’ of systems biology models [3–6], data integration and multi-scale modeling [7, 8]. ‘Reproducibility’, ‘interoperability’, and other important terms are explained in Table 1.

**Table 1:** Definition of terminology related to the reproducibility of systems biology models

Term	Definition in the context of systems biology models	References
Annotations	Descriptive information (metadata) used to characterize aspects such as authorship, literature sources, the meaning of model components (see ‘semantic annotations’), the terms of distribution (license), and others.	[10, 11]
Credibility	Evidence-based confidence in the predictive capability of a computational model for a context of use.	[12, 13]
Curation	Process that involves code verification and calculation verification of a model, generally by researchers not related to the original study.	[14]
Findability^a^	Characteristic of models (or data) that facilitates their discovery by humans and computers. Digital resources should have a unique and persistent identifier, and be indexed in a search engine or known repository.	[15, 16]
Interoperability^a^	Potentiality of a model code to be converted into other model implementation languages and run (executed) on different software. The use of relevant community standards (e.g. for implementation language and vocabularies) facilitates interoperability.	[15, 16]
Computational model = model code	Implementation of a mathematical model in software. Notably, the code contains numeric algorithms written in a machine-readable (programming) language, which aim to simulate the mathematical model through code execution.	[9, 10, 12, 13]
Mathematical model	The description of a conceptual model through correct mathematical equations, specific conditions and modeling data.	[12, 13]
(Model) plausibility evidence	Evidence supporting the choice of mathematical equations, model assumptions and parameters. It can be considered as primary (and not sufficient per se) credibility evidence.	[13]
Repeatability (also called, for simulation experiments, ‘methods reproducibility’^b^)	The capacity to reproduce the simulation results by using the original code in the same software. This requires access to the code, simulation specifications and any linked data. Published articles for computational models must ensure the model repeatability.	[17–19]
Replicability	Considered as a synonym of ‘Repeatability’ by Tiwari *et al.*, 2021.	[18]
Reproducibility (also called, for simulation experiments, ‘results reproducibility’^b^)	The capacity to reproduce the simulation results after writing the code de novo and/or by using different software, which implies ensuring the mathematical expressions are correct. In other words, it should be possible for researchers not related to the original study to reproduce its results, after reconstituting one or more parts of the study.	[17–19]
Reusability^a^	Potentiality of a model (model code and model description) and their provenance (authors and sources) to be understood and credited by both humans and computers.	[15, 16]
Semantic annotations	Computer-accessible description of model components.	[4, 11]
(Model) simulation	Reproduction of the model behavior in time (and/or space), under specific conditions. This is done through code execution and numerical integration performed by a solver.	[20]
Solver	Numerical algorithm or computational method used to solve a system of ODEs, starting from their initial state values. The solution is obtained iteratively for a predefined simulation time.	[21]
(Model) validation (or model verification)	Consistency between model predictions (simulated data) and real observations from relevant studies (observed, experimental data). This requires the use of experimental data not used at any step of the model development.	[12, 13, 22]
Calculation verification	Analysis of the capacity of a computational model (code) to solve the underlying mathematical model for the intended purposes.	[12, 13]
Code verification	Evaluation of the code and the mathematical model that it represents. Furthermore, the code should comply with MIRIAM guidelines, including annotations and standard formats if possible.	[12–14]

^a^One of the denominated ‘FAIR principles’. Briefly: ‘all research objects should be findable, accessible, interoperable, and reusable (FAIR) both for machines and for people’ (Wilkinson *et al.*, 2016; Jacobsen *et al.*, 2020). In this Table, we provide our interpretation of these principles as applied for systems biology models.

^b^In a broader research context, the term ‘reproducibility’ can be divided into: ‘methods reproducibility’: the methods should be sufficiently detailed to allow an exact repetition of procedures by others; ‘results reproducibility’: the ability for an independent study to obtain the same results, which requires methods reproducibility too; ‘inferential reproducibility’: the ability for an independent study to reach the same conclusions after either replicating or reanalyzing the original study [19, 23].

A systems biology model is a simplified representation of a biological system. The biological levels that may be represented range from molecules, cells, tissues and organs, to whole organisms and populations [2]. In this paper, we focus on a type of systems biology model that is of particular importance for the pharmacology and toxicology communities, physiologically-based pharmacokinetic (PBPK) models. PBPK models are one type of mechanistic quantitative systems pharmacology (QSP) models [7]. PBPK models represent the processes of absorption, distribution, metabolism, and elimination of chemicals—abbreviated as ‘ADME’, also known as pharmacokinetics, following exposure in humans or animal species [9]. These models allow linking the external exposure to a chemical with its absorption in the organism and the resulting internal exposure levels, represented by the time-dependent concentrations of the chemical—and/or its derived metabolites—in organs and tissues. PBPK models use ordinary differential equations (ODE) and include a realistic representation of relevant anatomical and physiological traits in the organism. They also take into account the biochemical characteristics of a substance (or chemical group) that impact its pharmacokinetics, and consider one or more external exposure scenarios [22, 24]. PBPK models are major tools in toxicological risk assessment of chemicals, where they can be combined with other types of models and data, and used for several regulatory applications [9, 22, 25–27].

The typical process of PBPK modeling is iterative and organized in several steps. The first step focuses on determining a clear purpose to develop a model, such as a precise scientific or regulatory question concerning an organism of interest. The second step is conducting a literature search, to have a global understanding of this organism and the chemical group of interest, and to identify the main exposure routes, processes, and interactions to consider for the research question. This information allows the creation of a ‘conceptual model’, which is a graphical overview of the processes to be represented. This stage also involves defining hypotheses about the system of interest. Importantly, to be able to simulate the model and make quantitative estimations, the conceptual model must further be developed by creating a ‘mathematical model’ and a ‘computational model’, the latter also often referred to as the ‘model code’ (see definitions in Table 1). The third step is then the definition of the mathematical model, which specifies the mathematical equations, conditions (in this case, exposure scenarios) and the data used for model development and validation [12, 13]. This step often involves mining literature and/or databases to find (and evaluate) available parameter values. The fourth step is coding the model by using numeric algorithms, a specific solver and numeric tools (Table 1). This way, the model is implemented in a machine-readable (programming) language. Upon code execution, the model solver performs a numerical integration to simulate the model’s behavior in time (and/or space), under predetermined simulation conditions [9, 10, 12, 13, 20]. Further steps include model evaluation through sensitivity analysis and model validation. Often an iterative approach is used to progressively refine the model to align with experimental observations and if needed update model parameters.

Computational studies must be ‘repeatable’, allowing other researchers to repeat the study by using the same source code and data [17], and ‘reproducible’, allowing other researchers to reproduce the model results after rebuilding the model or applying it using different software (see Table 1). This enables model peer-review and reuse.

To facilitate model reproducibility, good practices for encoding, annotating and describing a mathematical biological model exist in the literature. For example, Le Novere *et al.* [10] proposed the ‘Minimum information requested in the annotation of biochemical models (MIRIAM*)*’ standard. Globally, the MIRIAM recommendations highlighted the need for consistency between the model code and a single reference description of the mathematical model. MIRIAM specified the main information to include in the model description (equations, parameter values, initial condition values, simulation results), and annotation (model authors, model version and others). The MIRIAM guidelines were extended by the ‘Minimum Information About a Simulation Experiment (MIASE)’ guidelines [20]. Besides compliance with MIRIAM, MIASE requires a full description of the simulation conditions applied to obtain the model quantitative results, including the software used and information on any possible modification of parameter values before simulation.

In addition to the MIRIAM and MIASE guidelines for model documentation, specific good modeling practices for PBPK models also exist [22, 28]. Andersen *et al.* [22] highlighted the need to explain the model purpose and the hypothesis tested, present all the equations, values and units of parameters and variables (as in MIRIAM), state the model validity domain (e.g. species, age, physiological stage, chemical group), define the criteria applied for model evaluation (such as sensitivity and uncertainty analyses), describe the software used (as in MIASE) and make the code available upon request. Loizou *et al.* [28] mentioned the benefit for PBPK models of using standard encoding languages such as Systems Biology Markup Language (SBML) or CellML. Additionally, there are guidelines for documentation and evaluation of PBPK models developed for risk assessment purposes [9, 29].

In spite of existing guidance, the reproducibility of systems biology models is still a challenge. As an illustration, Tiwari *et al.* [18] analysed the reproducibility of 455 published deterministic ODE models, previously submitted to the BioModels repository [14]. Among these models, 49% were not directly reproducible, of which only approximately one quarter (12% of all models included in that study) were reproducible after manual correction, and three quarters (37% of the total) were not reproducible in any manner. More emphatically, Porubsky *et al.* [17] cited two independent studies by the BioModels and the Physiome repository groups, where the reproducibility of >1200 systems biology and physiology models was assessed. In those studies, >95% of the models evaluated were not reproducible (personal communication quoted by Porubsky *et al.* [17]). Tiwari *et al.* [18] identified several causes that limited model reproducibility, notably errors in the parameters and the initial conditions (missing values, incorrect units) and inconsistency of the model structure (errors in equations, missing terms, incorrect signs). In this context, to help estimate the reproducibility of systems biology models, Tiwari *et al.* [18] proposed a reproducibility scorecard, which focused on the most important information needed to ensure the model reproducibility, in line with MIRIAM and MIASE guidelines. Concerning the model code, the reproducibility scorecard pointed to the need for clarity, the importance of including a set of numerical results, the preference for using standard languages such as SBML and publishing the model in public repositories.

The low reproducibility of ODE-based biological models as reported by Tiwari *et al.* [18] may be an issue for PBPK models too. For example, Thompson *et al.* [30] reviewed 7541 PBPK models. As part of their review, the authors created and published a database containing the main descriptors (metadata) per model [31]. Within the database created by Thompson *et al.* [30], we found information for 1657 literature references based on their reference name and DOI. Of these, only 508 references (31%) were annotated as showing the full model equations either in the paper or in supplementary information. By contrast, the equations were not available for 528 references (32%). The model code was directly available only in 68 references (4.1%), generally as supplementary information (3.8%), with few exceptions where the code was published in BioModels or GitHub (0.3%). Out of 69 software platforms identified in the database by Thompson *et al.* [30], the preferred tools (% of all references) were: Simcyp (13.5%), ACSL (10.4%), MATLAB (7.2%), Berkeley Madonna (6.0%), and SIMUSOLV (5.4%). By contrast, the simulation software was not specified in 18% of all references. In summary, there is evidence that the reproducibility of PBPK models also needs to be improved.

**Figure 1:**
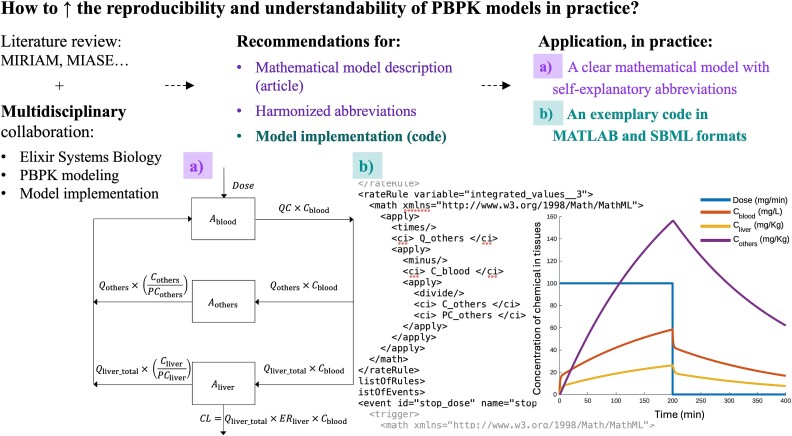
Graphical abstract of the research question and workflow in the current study. (a) (also shown with violet letters): information regarding notably the mathematical model description. The model diagram is shown (left), where, *A*_tissue_ and *C*_tissue_ represent the amount (mg) and the concentration of chemical in a tissue (mg/kg), respectively. *Dose* is the intravenous infusion of chemical (mg/min). *QC* is the cardiac output (L/min). *Q*_tissue_ is the blood flow (L/min) to a tissue. *PC*_tissue_ is the partition coefficient (L/kg) of the chemical between a tissue and the blood. *ER*_liver_ is the extraction ratio of the chemical in the liver (unitless). (b) (also shown with green letters): information regarding the model implementation (code). A screenshot with part of the SBML code is shown (center), and the model simulation figure (right). As an example, the same exposure scenario as by Upton *et al.* [32] was considered.

In this paper, we aim to: (i) help improve PBPK model reproducibility, and (ii) increase the comprehensibility of PBPK models for colleagues working in other fields of systems biology, given that each field requires a high level of specialization, to aid better communication and collaboration with other systems modelers. As summarized in Fig. 1, we provide a summary of recommendations for PBPK model reporting and implementation, in order to ensure reproducibility. This includes a proposal towards harmonizing abbreviations for PBPK model components. Finally and importantly, to illustrate all these recommendations, we present an original, reproducible, and flexible PBPK model code example (Fig. 1) alongside an example of the MATLAB code converted to SBML format using MOCCASIN—the conversion to SBML enables re-use of the model outside the MATLAB environment.

## Materials and methods

### Providing general recommendations for documentation and implementation of PBPK models

In general to ensure the reproducibility of a PBPK model, the model should be fully explained in and implemented through two main documents, which must be consistent and cite each other [10, 12, 13, 16, 20]:

1) A peer-reviewed journal article or, at minimum, a single reference description, where the mathematical model (Table 1) is described, and
2) The model code, which is the implementation of the mathematical model in software, including notably correct numeric algorithms and a specific solver, and which may require specific software libraries. The code allows model simulation in time, under specific conditions.

In order to develop and optimize both documents, we reviewed, summarized and completed recommendations for model description, documentation and annotation from major literature sources, notably:

- the MIRIAM set of rules [10], and ‘reproducibility scorecard’ [18];
- the MIASE guidelines [20];
- the ‘model reporting template’ for PBPK model assessment in a regulatory context by OECD (2021) [9] and the PBPK reporting Guidelines by the European Medicines Agency [33]. From these two sources, we include requirements for reporting of PBPK models in general. We do not include additional requirements for PBPK models created for specific regulatory purposes, which are explained elsewhere [9, 13, 33].

### Description of the PBPK model

**Figure 2:**
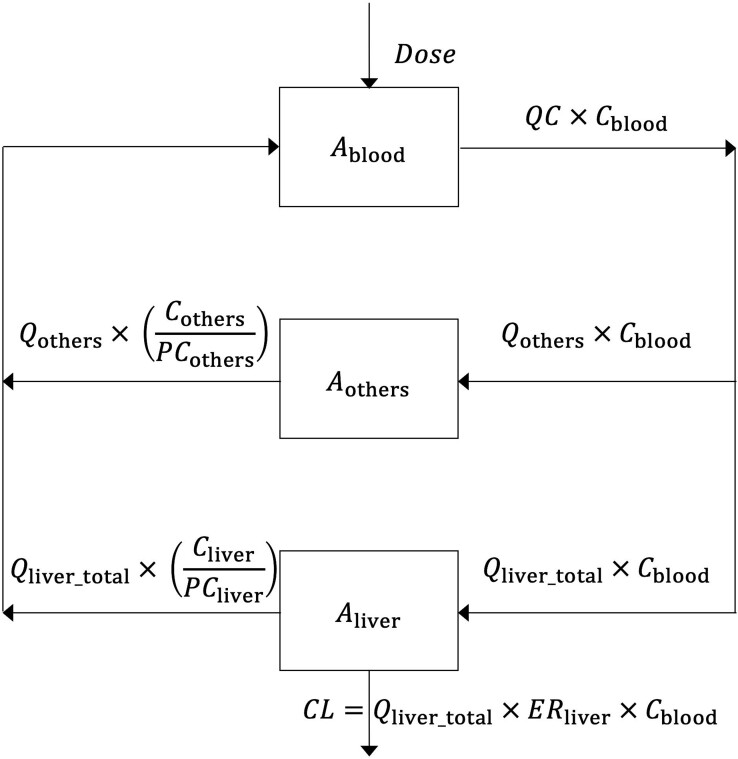
Diagram of the conceptual PBPK mathematical model, adapted from the conceptual model by Upton *et al.* (2016), with the differences explained in the Material and methods. *A* is the amount of the chemical in a given compartment (mg). *C*_blood_, *C*_liver_, and *C*_others_ are the concentrations of the chemical in the blood (mg/L), liver and other body tissues (mg/kg), respectively. *Dose* is the intravenous infusion of the drug (mg/min). *QC* is the cardiac output (L/min). *Q*_others_ and *Q*_liver_total_ are the blood flows (L/min) to other body tissues and to the liver, respectively. *PC*_others_ and *PC*_liver_ are the partition coefficients (L/kg) of the chemical in other body tissues and the liver, respectively. *ER*_liver_ is the extraction ratio of the chemical in the liver (unitless). The parameter values are shown in Tables 2 and 3.

We defined a simple, illustrative PBPK mathematical model for an adult man, adapted from a conceptual model by Upton *et al.* [32]. Similar to that model, our mathematical model contains three compartments. These are the blood, liver, and ‘others’ (meaning other body organs and tissues). Following intravenous exposure to the chemical, the distribution of the chemical from blood to liver and ‘others’ and vice versa (from liver and ‘others’ to blood) as well as the elimination of the chemical in liver are represented (see Fig. 2). As an example of an exposure scenario, we use the same dose and exposure time as in Upton *et al.* [32]: 100 mg/min for 200 min, followed by 200 min of depuration. We consider a hypothetical hydrophobic chemical that could globally have a higher accumulation in organs/tissues with higher total fat content (a simplifying hypothesis). The exposure scenario is an example and has no meaning in terms of saturable kinetics or chemical toxicity, which are not represented in the model. Differences between the mathematical model here and the conceptual model proposed by Upton *et al.* [32] are as follows:

- We use physiological parameter values representative for adult men (Table 2), as an example. For information, physiological parameter values for adult women are also shown in Table 2 and within the code.
- We represent intravenous administration of the chemical as an input into the blood compartment [34].
- We use classical flow-limited distribution equations, including partition coefficients of the chemical between each tissue and the blood (*PC*_tissue_, L/kg) [34, 35]. Under the simplifying hypothesis of a hydrophobic chemical with a higher accumulation in organs/tissues with higher total fat content, we selected random *PC*_tissue_ values higher than 1, because the average fat contents in the whole body (21.3% of BW in men, 32.7% of BW in women) and liver (7% of liver wet weight) are higher than the lipid content in blood (0.65%) [36, 37].
- The metabolism and elimination of the chemical through the liver (liver clearance, *CL*, mg/min) were grouped into one process, represented as follows [38, 39, 40]:

$$ CL={Q}_{liver\_ total}\times E{R}_{liver}\times{C}_{blood}, $$

where *Q*_liver_total_ is the total blood flow to liver (L/min), *ER*_liver_ the extraction ratio of the chemical in the liver (no units), and *C*_blood_ the concentration of the chemical in the blood (mg/L).

**Table 2:** Physiological parameter abbreviations, definitions, values, units, and sources in the proposed model example. The different values for adult men and women are shown. As an example, the values for men are used in the model

Abbreviation	Abbreviation in the code	Definition	Value	Units	Reference/s, source
*BW*	BW	Reference BW for European adult men	73 (men); 60 (women)	Kg	[36], p. 64
*W*_liver_	W_liver	Liver weight (Kg) for an adult person	0.026*BW (= 1.90 in men; = 1.56 in women)	Kg	Calculated as being ~2.6% of the body weight, both in men and women [37], p. 429
*W*_blood_	W_blood	Total blood weight in an adult person	5.3 (men); 3.9 (women)	Kg	We use the same value as for *V*_blood_ (see below). In fact, a mass-to-volume conversion may not be needed in PBPK models for most organs, with density values close to 1 g/mL (1.02–1.06) [37], p. 432. This is also the case for blood tissue, with average measured densities around 1.06 [36, 45] and 0.99 g/mL [46]
*W*_others_	W_others	Weight of other tissues (Kg)	*BW*-*W*_liver_-*W*_blood_ (= 65.8 in men; 54.54 in women)	Kg	Calculated as the difference between the body weight and the weights of liver and blood
*QC*	*QC*	Reference cardiac output (L/min) for an adult person	6.5 (men); 5.9 (women)	L/min	[36], p. 139
*Q*_liver_total_	Q_liver_total	Reference value of total blood flow to liver (L/min), including flows from the portal vein and hepatic artery, for an adult person	In men: 0.255*QC = 1.66 In women: 0.270*QC = 1.59	L/min	The total blood flow to the liver is calculated as being ~25.5% (in men) and 27% (in women) of the cardiac output [36], p. 142
*Q*_others_	Q_others	Blood flow to the rest of the body (L/min)	*QC*-*Q__liver_total_ (*=4.84 in men; =4.31 in women)	L/min	Calculated as the difference between *QC* and *Q*_liver_total_
*V*_blood_	V_blood	Reference value of total blood volume (L) for an adult person	5.3 (men); 3.9 (women)	L	[36], p. 139

The abbreviations, definitions, values, units, and sources of physiological and pharmacokinetic parameters in the model are explained in Tables 2 and 3. The model variables are shown in Table 4.

**Table 3:** Pharmacokinetic and exposure-related parameters: abbreviations, definitions, values, units, and sources in the proposed model example

Abbreviation	Abbreviation in the code	Definition	Value	Units	Reference/s, source
*ER*_liver_	ER_liver	Extraction ratio of chemical in the liver, grouping metabolism and elimination processes (Emond *et al.*, 2010)	0.70	Unitless	ER is a ratio and its value should be between 0 and 1, depending on the chemical. We select a random value, 0.7, which corresponds to a high clearance scenario in the animal model by MacLachlan [38].
*PC*_*_*liver_	PC_liver	Partition coefficient of chemical between the liver and the blood	1.5	L/kg	Random values. We consider a hypothetical hydrophobic chemical that could globally have a higher accumulation in organs/tissues with higher total fat content (as a very simplistic example). The total fat content is higher in the whole body (average values of 21.3% of BW in men; 32.7% of BW in women) and in the liver (7% of liver wet weight), compared to the lipid content in blood (0.65%) [36, 37]. Therefore, for our hypothetical chemical, we propose random PC values in ‘others’ and in liver >1
*PC*__others_	PC_others	Partition coefficient of chemical between ‘others’ (other tissues) and blood	3	L/kg	
*Dose*	*Dose*	Drug dose, administered intravenously	100	mg/min	Exposure scenario as in Upton *et al.* [32], including the dose, exposure time, and differing in the exposure route (intravenous in our model)
*Time*_start_simul_	Time_start_simul	Starting time for the simulation	0	Min	
*Time*_end_simul_	Time_end_simul	Time at which the simulation ends	400	Min	
*Time*_start_expos_	Time_start_expos	Time at which the chemical exposure begins	0	Min	
*Time*_end_expos_	Time_end_expos	Time at which the chemical exposure ends	200	Min	

**Table 4:** System of variables and differential equations in the MATLAB code created here, including the initial values for these variables

Equation number	Equation	
Variables		
Flow of chemical infusion (dosing; mg/min)		
Equation 1	If time < *Time*_start_exp_ or time > *Time*_end_exp_: Dosing = 0	
	Otherwise Dosing = *Dose*	
Concentration of the chemical in blood (mg/L), liver and others (mg/kg), respectively		
Equation 2	${C}_{blood}=\frac{A_{blood}}{V_{blood}}$	
Equation 3	${C}_{liver}=\frac{A_{liver}}{W_{liver}}$	
Equation 4	${C}_{others}=\frac{A_{others}}{W_{others}}$	
ODEs		
Net flow of chemical to the blood, liver and others (mg/min), respectively		
Equation number	Equation	Initial state value, at time = 0
Equation 5	$\frac{d\left({A}_{blood}\right)}{dt}={Q}_{liver\_ total}\times \left(\frac{C_{liver}}{P{C}_{liver}}-{C}_{blood}\right)+{Q}_{others}\times \left(\frac{C_{others}}{P{C}_{others}}-{C}_{blood}\right)+ Dosing$	0 mg
Equation 6	$\frac{d\left({A}_{live r}\right)}{dt}={Q}_{live{r}_{total}}\times \left({C}_{blood}-\frac{C_{live r}}{P{C}_{live r}}\right)-{Q}_{live{r}_{total}}\times E{R}_{live r}\times{C}_{blood}$	0 mg
Equation 7	$\frac{d\left({A}_{others}\right)}{dt}={Q}_{others}\times \left({C}_{blood}-\frac{C_{others}}{P{C}_{others}}\right)$	0 mg

Where *A* is the amount of chemical in a given compartment (mg). *C*_blood_, *C*_liver,_ and *C*_others_ are the concentrations of the chemical in the blood (mg/L), liver, and other body tissues (mg/kg), respectively. *V*_blood_ is the blood volume (L), *W*_liver_ and *W*_others_ are the weights of liver and other body tissues (kg), respectively. *Q*_others_ and *Q*_liver_total_ are the blood flows (L/min) to other body tissues and to the liver, respectively. *PC*_others_ and *PC*_liver_ are the partition coefficients (L/Kg) of the chemical in other body tissues and the liver, respectively. *Dose* is the intravenous infusion of the drug (mg/min). *ER*_liver_ is the extraction ratio of the chemical in the liver (unitless). The parameter values are shown in Tables 2 and 3. The integration of the differential equations was conducted using the ode23s solver in MATLAB.

### MATLAB implementation

Following the definition of the mathematical model, we implemented it in software. We wrote an original code in MATLAB. The objective of this implementation was to simulate the model behavior with time. Within the code, we specified all the model parameters, the exposure scenario, and the model variables (see Tables 2–4), as executable algorithms. The model variables to be integrated were represented as a system of 3 differential equations. We considered for example that the initial value of these variables (at time 0 of simulation) was 0 (random value; Table 4). For integration, we used the ode23s solver in MATLAB as it can robustly integrate stiff systems of equations, i.e. those where the solution components may change slowly for an extended period of time. The model was created and simulated using MATLAB R2019b and MATLAB Online (basic) version.

### Code conversion from MATLAB to the Systems Biology Markup Language (SBML)

For interoperability, we converted the MATLAB code to SBML, by using the ‘Model ODE Converter for Creating Automated SBML Interoperability (MOCCASIN)’ tool [41, 42], version 1.3.0 [43]. This conversion was not straightforward and required adaptations on the MATLAB code, whereby we had to replace the function calls by the function formulas. Following this, MOCCASIN converted the MATLAB code to a preliminary SBML code. Then, we simulated the preliminary SBML code in COPASI (version 4.44) [18, 44]. For this, a few further changes were needed: (i) harmonization of the names of variables (y__1 was replaced by ‘integrated_values_1’, same for y__2 and y__3); (ii) replacing the conditions for dosing with an event; and (iii) manual introduction of the units for parameters and the simulation time. Finally the whole model was exported again to SBML.

## Results

We start this section with a summary of recommendations aiming to ensure PBPK model reproducibility. As an illustration, we applied these recommendations during the development and implementation of the PBPK model code described in this manuscript (see Fig. 1). The original model code is explained below.

### General recommendations for documentation and implementation of physiologically-based pharmacokinetic models

The recommendations for PBPK model reporting and implementation are shown in Tables 5–7 and in Supplementary Information (SI), Tables S1 and S2.

**Table 5:** Recommended model^a^ annotations (descriptive information) and naming of model components to be included in the journal article^b^ and within the code^c^. The underlined text denotes the additional proposals that we include in the current article for PBPK models, which may also be of interest for other systems biology models

Type of information	Recommendations and explanation	References
Model identification and files	Name of the model. Clear identification of two main files: the journal article^b^ and the model code^c^, which must be consistent and mutually cite each other (ideally, cite the unique persistent identifier of each other). Identification of other files (e.g. databases) and resources associated to the code, through citations and links.	[9, 10, 16, 47–49]; this article
Model authors	Names (and ORCIDs) of the authors of the journal article^b^, with contact details. Names (and ORCIDs) of the authors of the model code^c^, with contact details. Affiliations and funding sources.	
Code sources	Citations of the sources of both the mathematical model and the computational model. For example, if a model code is reused as the base to develop another model code, the resulting code and its article must: (i) cite the source code (and version) and its journal article, and (ii) explain the differences between the source code and the derived code. See license (below). Explicit mention to any artificial intelligence tools used to either develop, reuse, or modify a model code.	
Model code^c^ version	Dates of creation and last modification (version) of the model code. Importantly, version control is automatic in public repositories and sharing platforms, such as BioModels and GitHub, respectively.	
License	License CC BY 4.0 has been recommended for research work [47, 48].	
Naming of model components	Clear and unambiguous names for the model components (in the absence of standard names in a given field). One should prioritize self-explanatorynames. It is essential to ensure that the model component names are the same within the code^c^ and in the journal article^b^ (consistency).	[10, 18]; this article

^a^Although the focus of the current paper is on PBPK models, most recommendations in this Table are applicable to systems biology models in general, as well as PBPK models.

^b^This refers to the peer-reviewed journal article or single reference description, where the mathematical model is described [10].

^c^See definitions in Table 1.

**Table 6:** Recommendations for mathematical model^a^ description in the journal article^b^. the specific requirements for PBPK models are given in bold. The underlined text denotes the additional proposals that we include in the current article for PBPK models, which may also be of interest for other systems biology models.

Type of information	Recommendations and explanation	References
Model scope	Model purpose. **Summary of the model ‘applicability domain’, including: species, sex, age/physiological stage, specification whether the model is ‘generic’ (it can be easily adapted to be used for different chemicals) or ‘specific’ for a given chemical group, characterization of chemical/s, exposure scenarios**.	[9, 10, 18, 22, 28, 33, 50], this article
Conceptual and mathematical model	Mathematical representation of model processes, **considering: physiological features, physicochemical properties and pharmacokinetic processes important for the chemical/s of interest**. Model assumptions. **Clear and complete diagram of the conceptual model, where the model structure and processes are shown graphically**. The conceptual model helps the reader visualize and understand the mathematics underlining a model. Thus,the conceptual model is important and must be consistent with the (latest version of the) mathematicalmodel.	
Parameterization, including units and sources	Values/ranges of parameters, with correct units and citation of sources and/or estimation methods. The parameter information should be shown within Tables in the article. Initial values (**at the beginning of the simulation) for the concentration of chemical in physiological** compartments, with sources / estimation methods and units.	
Identification of major files with the article	Clear identification of the model code^c^ and explanation where the code can be found. A set of quantitative results obtained by model simulation, with a description of the simulation conditions (Table 7).	[9, 18, 20, 33],

^a^Although the focus of the current paper is on PBPK models, most recommendations in this Table are applicable to systems biology models in general, as well as PBPK models. Recommendations specific for PBPK models are shown with bold text.

^b^This refers to the peer-reviewed journal article or single reference description, where the mathematical model is described [10].

^c^See definitions in Table 1.

**Table 7:** Model code^a^^,^^b^ content, structure and understandability, model implementation language, and dissemination. Specific requirements for PBPK models are in bold, and the underlined text denotes the additional proposals that we include in the current article for PBPK models, which may be of interest for other systems biology models too.

Type of information	Recommendations and explanation	References
Major elements (content)	Numeric algorithms and solver, values of parameters (with annotation of the units and sources), initial values of model variables (with annotation of the units and sources), specific software libraries, if needed, clear identification of the journal article, as specified in Table 5.	[9, 10, 18, 20]
Provenance and understandability	Annotations specified in Table 5, Other annotations, such as text definitions of model components, sources and others, in order to facilitate the code understandability for the users.	
Model simulation results	A set of quantitative simulation results. These data, produced by execution of the model code^b^, must be provided with the journal article^c^ in order to facilitate later evaluations of model reproducibility. If the model code is publicly available, ideally the code execution can automatically create a Table ofquantitative simulation data (as in the code proposed as an example in this paper).	[9, 18, 20, 33]; this article)
Description of model simulation conditions	Specification of the software environment and version, if needed, specific software libraries, model solver, simulation conditions, including the: **simulation time (for ODE models such as PBPK models), exposure scenarios**, any changes in parameter values or state conditions, any performed post-processing normalization of results.	
Implementation language and model code dissemination	Implementation in software and dissemination of systems biology models, ideally, in (community-) standard formats such as SBML, CellML and others. Publication of PBPK model codes written in MATLAB, R, and other languages (Hack *et al.*, 2020) in the programming language where they were originally created. Ideally, dissemination of the code also in SBML format (after conversion). Deposition of the code and associated files via a reliable open model repository. We recommend to check license terms. License: here, we have selected CC BY 4.0 (Margoni and Tsiavos, 2019; OpenAIRE, 2024; Table 5).	[9, 10, 18, 28, 47, 48]; this article)

^a^Although the focus of the current paper is on PBPK models, most recommendations in this Table are applicable to Systems Biology models in general, as well as PBPK models. Recommendations specific for PBPK models are shown with bold text.

^b^The model code or ‘computational model’ is the implementation of the mathematical model in software [9, 10, 12, 13].

^c^This refers to the peer-reviewed journal article or single reference description, where the mathematical model is described [10].

In the first place, we show the main model annotations (descriptive information) to be included both in the journal article and within the code (Table 5). This includes notably a clear identification of: the model (name and version), authors (including ORCIDs), the sources for the model and code, and license.

In the second place, we summarize the information to describe the mathematical model in the journal article (Table 6). Here, we refer in particular to the model purpose and applicability domain, structure, main assumptions, the model component values (with sources and units), and a set of quantitative results obtained by simulating the model.

Importantly, in Table 7, we provide recommendations for model implementation. For instance, we list the major executable elements within the code (numeric algorithms, solver, and numeric values of model components), important annotations, and others. The execution of the model code under specified simulation conditions must produce quantitative results, which should be published to facilitate reproducibility checks.

Furthermore, to make PBPK models more comprehensible, the naming and abbreviations of model components is important. In supplementary information (SI, section B), we provide general recommendations for parameter naming. Further, we show examples of abbreviations used in the literature for important processes and model components. For each process, we propose one or two abbreviations which seem the most clear and self-explanatory to us (SI, Table S1).

Finally, although not the focus of this work, other analyses for model evaluation, such as sensitivity analysis and model validation are mentioned in SI, Table S2.

### Writing an original, reproducible, and reusable PBPK model code in MATLAB and SBML formats

We have created an original PBPK model code in MATLAB and SBML formats. This code adheres to the recommendations for PBPK model reproducibility summarized in Results. The code and the numeric simulation results are available in Zenodo (https://doi.org/10.5281/zenodo.13838845).

Briefly, the MATLAB code starts with the model annotations shown in SI, Table S3. Next, we include all the model parameters (abbreviations, values, units, definitions, sources) and the exposure scenarios, as defined within Tables 2 and 3. We specify the initial values of the model variables, as in Table 4. This is followed by the definition of the model solver (ode23s function solver in MATLAB) and the system of differential equations (Table 4). In the last part of the code, we show the literature references cited within the code.

When the code is executed in MATLAB, the model solver integrates the system of differential equations during the simulation time, starting from the initial values of the model variables, and taking into consideration all the parameter values and the chemical exposure scenario. The model simulates the time-dependent concentrations of the chemical in blood, liver, and *others*. By execution of this code, the model simulation results are automatically provided both as a figure (SI, Fig. S1, left panel) and in the form of an Excel Table, also available in Zenodo. This Table is important since it gives other researchers the possibility to independently check the reproducibility of the model results.

The SBML code includes all the quantitative information (parameter values, equations, initial values, dose events) needed to simulate the model. To execute a SBML code, tools such as COPASI and JWS online may be used [44]. When the SBML code is executed in COPASI, the simulation results (SI, Fig. S1, right panel) are the same as with MATLAB (SI, Fig. S1, left panel).

## Discussion

Our work contributes to making PBPK models more reproducible and understandable. We reviewed the literature on proposed practices to increase the reproducibility of systems biology models in general and PBPK models in particular. Some published recommendations are globally applied in PBPK models, but some aspects from minimal information guidelines such as MIRIAM and particularly MIASE are not systematically applied in this field. These aspects include: model annotation, the description of the simulation conditions, the publication of numeric simulation results (to facilitate reproducibility assessment), the definition of license terms and the publication of the code. Reproducibility issues in many PBPK models include notably not sharing the code and not showing the full model equations [30, 31].

An important originality of our study is its applied perspective. Cronin *et al.* [49] discussed the conceptual application of FAIR principles (Findable, Accessible, Interoperable, Reusable) to *in silico* models for toxicology, including PBPK models. Tatka *et al.* [51] reviewed recommendations and relevant standards to increase the credibility of systems biology models for their context of use. We also review existing recommendations from the literature. Nevertheless, we focus on recommendations which help increase the reproducibility and comprehensibility of systems biology models and particularly of PBPK models. We illustrate these recommendations through the explanatory model code.

The recommendations summarized in Tables 5–7 are meant to ensure a complete and clear description of the mathematical model in the publication and a consistent model implementation. For future PBPK models, these recommendations are directly applicable during model development, description and publication. For models which are already published, the recommendations in Tables 5–7 can help detect potential missing / unclear or inconsistent information in those models, which should be then corrected by the model authors or by expert curators. Our recommendations could also be used as reproducibility criteria to select models for further development and reuse. We recommend reproducibility checks on published PBPK models start with a verification of the following aspects: (i) the mathematical equations for the model processes are shown and the abbreviations in the equations are briefly defined (e.g. Table 4 with legend), and (ii) the model parameter values are shown with their units, sources, and abbreviations. Other reproducibility recommendations could be checked next.

We have created a clear and reproducible PBPK model and code, which is available in Zenodo. Many PBPK models in the literature do not have an associated model code available. We believe that our work helps understand the importance of sharing the model code for reproducibility. A key point to consider for model code publication is the existence of different license options. For example OpenAIRE, a major actor of the European Open Science Cloud, has recommended research work be licensed under CC BY 4.0 [47, 48]. Under CC BY 4.0, the work can be reused by others, who must give appropriate credit to the creators and indicate if they (the users) have made changes to the original material [52]. Original model code, such as the one we present here, is an important scientific result which requires expertise both on the type of model represented and on programming [53]. For these reasons, we decided to publish the PBPK model code under CC BY 4.0. Nevertheless, further exploration on license choices for future models would be beneficial.

As a limitation of our work developing an exemplar reproducible model code, we encoded the model in commercial software. We selected MATLAB for this based on its wide application in this field, its flexibility (which permitted us creating the code from scratch), human-readability, and our own experience with this software environment. In reality, our recommendations are independent of the software choice, since we focus on ensuring (i) the completeness and clarity of the model description and (ii) the consistency between the mathematical and computational model. So far, no standard format has been generalized in the PBPK modeling field. Further, many software options exist for PBPK model implementation [53]. In the PBPK model review database by Thompson *et al.* [30, 31], up to 69 software tools were identified in the reviewed models. Of those, Simcyp, ACSL, and MATLAB were the three most common tools. Further, the results of an online survey to modelers in QSP confirmed the popularity of MATLAB, which was the preferred software tool among participants to that survey (*N* = 105) [54]. This may be in part explained by a wide application of these models for regulatory purposes and in commercial settings, where a recognized commercial platform like MATLAB may be preferred.

Recognizing the need to share models on a non-proprietary platform we have also created a standard version of the code, written in SBML. SBML is a standard format widely used in systems biology to facilitate model reuse and interoperability [6, 14, 18, 51, 55]. This conversion was not straightforward and required some adaptations on the code (see Material and methods). On the one hand, this illustrates that there are constraints that can apply to a PBPK code in MATLAB if it is intended subsequently to share it more openly. On the other hand, MATLAB scripts are easy to annotate (as comments) and to understand, whereas SBML and other standard formats such as CellML [51] are important for model interoperability and reuse, but not user-friendly when it comes to checking the code. As a direction for future research, further development of tools / methods to ease conversion to PBPK model code (such as the example we provide) from MATLAB and other software to SBML or other standard formats would be desirable.

For our example code we did not conduct sensitivity analysis, where the sensitivity of the model results to variations in parameter values is analyzed, or model validation, where model simulations are compared with experimental data not used at earlier stages of the model development. These verification steps are important for PBPK models developed to be applied in chemical risk assessment or for similar purposes. However, here the objective was to help improve the reproducibility of PBPK models. For this, we focused principally on the stages of model development and implementation, where the main reproducibility issues in biological models arise. We verified the biological plausibility of the model, by carefully selecting physiological parameter values from the literature, and by ensuring that the body weight was the sum of the weights of all tissues and that the cardiac output was the sum of blood flows to all tissues. We conducted ‘code verification’ and ‘calculation verification’ (see Table 1), by ensuring the consistency between the mathematical model and the model code, and checking the quantitative simulation results. Hence, we confirm that the PBPK model developed here is mathematically correct and biologically plausible and the code allows simulation of the mathematical model.

Our code is open and flexible and facilitates potential modifications by the users for re-use. As a case study, we apply the model for an adult man. To simulate the model for a woman, the user can replace in the code the physiological parameter values for men by those for women, which we show in Table 2 and as comments within the code. Other examples of potential modifications on the code to represent different mathematical models are discussed in SI, section C. Following modifications on the code, particular attention would be needed to ensure that the mathematical model description and the model code are consistent, and to evaluate and validate the resulting model for its purpose.

Finally, the use of consistent and clear abbreviations for all the model components is crucial to increase the understandability of PBPK models. Whenever possible, controlled vocabularies and semantic annotations should be used [5, 11, 20]. However, to our knowledge, there are no such resources for many PBPK model components (e.g. parameters). In this context, it is essential to define all the abbreviations and use them consistently in the article and within the code. As a first step towards harmonization, we propose a list of abbreviations for PBPK models (SI, Table S1). We have prioritized examples of abbreviations that are frequently used in this field. In general, self-explanatory abbreviations are more understandable and would also be preferred. For the same reason, as subscripts to specify the tissues / organs (where needed), we recommend using their full names. We have selected abbreviations for several major PBPK model components, but this list is not exhaustive and does not consider all processes which can be represented in PBPK models. As a future development, it would be valuable to create a dynamic list where new abbreviations for other model components and processes are added, the proposed abbreviations discussed with the broad community of model developers and users, and harmonized. In the end, a standardized list would permit that new PBPK models apply the same abbreviations for the same processes, which would be an important progress towards ensuring PBPK model comprehensibility and reuse.

## Conclusion

In summary, we believe our work helps towards convergence of reproducibility practices in the PBPK modeling and other systems biology fields, from an applied perspective. We illustrate how to apply the recommendations step by step, from the creation of a conceptual mathematical model to the model implementation and publication. Further, by converting the code from MATLAB to SBML, our work can be easily visualized by modelers in other systems biology fields too.

One aspect which warrants further investigation is the practical application of standards to PBPK models. Standards are developed by the systems biology community, coordinated by the Computational Modeling in Biology Network (COMBINE), to facilitate model interoperability, reusability and integration [4, 6, 8, 56]. Here, we emphasize the use of standards where they exist. As an example, we have converted the explanatory MATLAB code to SBML, by using MOCCASIN. This conversion was not straightforward and some manual adaptations on the code were needed. Therefore, we recommend developing more conversion tools to allow an automatic conversion of PBPK codes from different software environments to SBML. Other examples of standards are COMBINE archives and the Simulation Experiment Description Markup Language (SED-ML) [4]. COMBINE Archives allow to organize in a single ‘container’ all the model files available in standard formats [44, 56]. SED-ML permits to create machine-readable descriptions of the simulation conditions and their results [57].

In our work, the model files are identified (Table S3) and available (see Data availability statement). We describe the simulation conditions in Tables 2–4 and the numeric simulation results are also provided. The applicability and added value of standards such as COMBINE Archives and SED-ML for PBPK models should be explored. As explained by König *et al.* [5], the suitability and potential adaptations of standards for specific systems biology fields should be analyzed and determined through cross-domain collaborations.

To conclude, this work is the result of a multidisciplinary collaboration between modelers and researchers in PBPK modeling and in other systems biology fields, with a major involvement of the ELIXIR Systems Biology Community. More multidisciplinary collaborations should promote further harmonization of good modeling practices in both fields.

**Key Points**

- We review and summarize literature recommendations aiming to ensure the reproducibility of PBPK and systems biology models.
- We illustrate our recommendations by creating and publishing an example of reproducible and understandable PBPK model code in MATLAB, which we have converted to SBML.
- We propose a list of self-explanatory abbreviations for PBPK model components, as a first step towards a dynamic list of harmonized abbreviations.
- Our work helps increase the reproducibility of PBPK models, make them more comprehensible, and harmonize good modeling practices in systems biology.

## Supplementary Material

Revised_SI_Dominguez-Romero_et_al_bbae569

## Data Availability

The original PBPK model code in MATLAB and SBML formats, and the numeric simulation results in MATLAB are available in ZENODO, under CC BY 4.0 license (https://doi.org/10.5281/zenodo.13838845; the current manuscript should be cited).
