# Supplementary material for: Making PBPK models more reproducible in practice
Source: Brief Bioinform. 2024 Nov 4;25(6):bbae569. doi: 10.1093/bib/bbae569 (PMC11533111; doi:10.1093/bib/bbae569)
Supplement: Revised_SI_Dominguez-Romero_et_al_bbae569 [file revised_si_dominguez-romero_et_al_bbae569.docx]

Supplementary Information (SI) to the article:

Making PBPK models more reproducible in practice

Elena Domínguez-Romero^1,2*^, Stanislav Mazurenko^3,4^, Martin Scheringer^2^, Vítor A.P. Martins dos Santos^5^, Chris T. Evelo^1^, Mihail Anton^6,7,#^, John M. Hancock^8,#^, Anže Županič^9,#^, Maria Suarez-Diez^10,#^.

^1^ Maastricht University, Department of Bioinformatics - BiGCaT, Maastricht, The Netherlands

^2^ RECETOX, Faculty of Science, Masaryk University, Brno, Czech Republic

^3^ Loschmidt Laboratories, Department of Experimental Biology and RECETOX, Faculty of Science, Masaryk University, Brno, Czech Republic

^4^ International Clinical Research Center, St. Anne’s University Hospital Brno, Brno, Czech Republic

^5^ Laboratory of Bioprocess Engineering, Wageningen University & Research, Wageningen, 6708 PB, The Netherlands.

^6^ ELIXIR, Wellcome Genome Campus, Hinxton, Cambridgeshire, CB10 1SD, UK

^7^ Department of Life Sciences, NBIS, SciLifeLab, Chalmers University of Technology, Gothenburg SE-412 96, Sweden

^8^ Institute of Biochemistry and Molecular Genetics, Faculty of Medicine, University of Ljubljana, Zaloška 4, 1000 Ljubljana, Slovenia

^9^ National Institute of Biology, Department of Biotechnology and Systems Biology, Večna pot 121, 1000 Ljubljana, Slovenia

^10^ Wageningen University & Research. Agrotechnology and food sciences. Stippeneng 4, 6708 WE Wageningen

*Corresponding author

E-mail: [elena.dominguez-romero@maastrichtuniversity.nl](mailto:elena.dominguez-romero@maastrichtuniversity.nl)

# last authors for this work

## Table of contents

[A. Supplementary Tables and Figures 3](#_Toc178344349)

[Table S1: recommended abbreviations (with subscripts) for major pharmacokinetic and physiological parameters and processes in PBPK models. 3](#_Toc178344369)

[Table S2: steps for model verification and curation. The specific requirements for PBPK models are given in bold. 7](#_Toc178344370)

[Table S3: important annotations (descriptive information) for the model developed in the current article. The annotations follow the guidelines provided in Table 5 in the manuscript……………………………………………………………….……………….8](#_Toc178344371)

[Figure S1: Simulated concentrations of chemical in blood (mg/L), liver and other tissues (mg/Kg), obtained by simulation of the proposed model code in MATLAB (left), and by execution of the SBML code in COPASI (right). 10](#_Toc178344395)

[B. Recommendations for naming and abbreviations of model components, notably parameters and state variables 11](#_Toc178344350)

[C. Code re-use: examples of potential modifications on the code 13](#_Toc178344351)

[References 14](#_Toc178344352)

## Supplementary Tables and Figures

Table S1: recommended abbreviations (with subscripts) for major pharmacokinetic and physiological parameters and processes in PBPK models.

| **Recommended abbreviation^a^** | **Other examples of abbreviations in PBPK models ^b^** | **Definition** | **Units** | **References** |
| --- | --- | --- | --- | --- |
| ***Pharmacokinetics: clearance, metabolism and elimination parameters*** | | | | |
| ***CL*** | *CL*_p_ (for plasma clearance); *CL*_b_ (for blood clearance) | Clearance from the systemic circulation | Volume Time^-1^ KgBW^-1^ | (Buxton & Benet, 2011; EMA, 2018; OECD, 2021) |
| ***CL*_int_** |  | Intrinsic clearance | Volume Time^-1^ ; other variations (e.g. μmol min ^-1^ mg protein ^-1^) | (Bessems et al., 2014; EMA, 2018; OECD, 2021; Stader et al., 2019) |
| ***CL*_kidney_** | *CL*_renal_ ; *CL*_r_ ; *Cl*_uri_ | Renal clearance | Volume Time^-1^ KgBW^-1^ | (Bessems et al., 2014; Buxton & Benet, 2011; Emond et al., 2010; Stader et al., 2019) |
| ***CL*_liver_** | *CL*_h_ ; *CL*_H_ ; *CL*_hepatic_ | Hepatic clearance | Volume Time^-1^ KgBW^-1^ | (Bessems et al., 2014; Buxton & Benet, 2011; EMA, 2018) |
| ***ER*_liver_** | *E*_H_ ; *E* | Extraction ratio / coefficient in the liver | Dimensionless | (Buxton & Benet, 2011; Emond et al., 2010) |
| ***GFR*** |  | Glomerular filtration rate (kidney) | Volume Time^-1^ | (Bessems et al., 2014; OECD, 2021) |
| ***V*_max_** | *v*_m_ | Maximal metabolic / elimination rate | Mass Time ^-1^ | (Bessems et al., 2014; Buxton & Benet, 2011; OECD, 2021) |
| ***K*_m_** |  | Michaelis-Menten constant, concentration at half the maximal elimination rate | Mass Volume ^-1^ | (Bessems et al., 2014; Buxton & Benet, 2011; OECD, 2021) |
| ***K*_urine_** | *k*_urine_chemical_ | Urinary elimination rate | Time^-1^ ; variations  (e.g. h^-1^ kg^-0.25^) ^d^ | (Chen et al., 2024) ; (Loccisano et al., 2011)^d^ |
| ***K*_degrad_ ; *K*_metab_process_** | *K*_deg_ ; *k*_deg_ ; *CL*_T int_ (where *“T”* was replaced by the first letter of the tissue name, e.g. *CL*_L int_ for metabolism in liver); *k*_glucuronidation_ (for metabolism by glucuronidation). | Rate of metabolism / degradation | Time^-1^ | (Chen et al., 2024; EMA, 2018; Stader et al., 2019) |
| ***Pharmacokinetics: Exposure and absorption*** | | | | |
| **Dose** |  | Dose of chemical | Mass KgBW^-1^ | (Renwick, 2001) |
| ***P*_app_route_** | *P*_app_ ; *K*_skin_absorption_ (dermal) ; *K*_p,app_ (dermal only) ^e^ | Apparent permeability coefficient for oral, dermal or inhalation absorption | Length Time^-1^ | (Bessems et al., 2014; Chen et al., 2024; OECD, 2021) |
| ***K*_abs_** | *k*_a_ ; *K*_a_ ; | Overall first order absorption rate constant | Time-1 | (Bessems et al., 2014; EMA, 2018) |
| ***Pharmacokinetics: Distribution*** | | | | |
| ***A*_tissue_** | *A*_t_ | Amount of chemical in a tissue / organ | Mass | (Emond et al., 2010) |
| ***dA*_tissue_ *dt*^-1^** | *dA*_t_ *dt*^-1^ | Time derivative for the amount of chemical in a tissue / organ | Mass Time ^-1^ | (Emond et al., 2010) |
| ***C*_tissue_** | *C*_t_ ; *C*_A_ or *C*_a_ (for arterial concentration) ; *C*_V_ or *C*_b_ (for venous concentration); | Concentration of chemical in a tissue / organ | Mass Volume ^-1^ | (Buxton & Benet, 2011; Emond et al., 2010) |
| ***f*_u_** | *F*_u_ ; *fu*_chemical_ ; *fu*_PL_ ; *Free* | Fraction unbound (free fraction, not bound to proteins) in blood or plasma | Dimensionless | (Bessems et al., 2014; Chen et al., 2024; EMA, 2018; Loccisano et al., 2011; OECD, 2021; Stader et al., 2019) |
| ***PC*_tissue_** ; *PC*_tissue_blood_ ; *PC*_tissue_plasma_ | *P*_tissue_plasma_chemical_ ;  *K*_t:b_ ; *K*_tb_ ; *K*_tissue:blood_ ; *PC*_tb_ ; *P*_tb_ ; *PT* or *Pt* (where *“T”* or *“t”* were replaced by the first letter/s of the tissue name, e.g. *PL or Pli* for liver); | Partition coefficient of a chemical between a tissue and the blood (or between a tissue and the plasma) | Dimensionless;  (Volume Mass^-1^) ^f^ | (Bessems et al., 2014; Chen et al., 2024; Emond et al., 2010; Loccisano et al., 2011) |
| *PC_u__*_tissue_plasma_ | *K*_tpu_ | Tissue : plasma water partition coefficient for the unbound fraction of the chemical | Dimensionless | (Bessems et al., 2014) |
| *PC*_tissue_air_ | *K*_t:a_ ; *K*_ta_ ; | Tissue : air partition coefficient | Dimensionless | (Bessems et al., 2014) |
| *PC_blood_*__air_ | *K*_b:a_ ; *K*_ba_ ; *K*_blood:air_ ; *P*_plasma_air_chemical_ | (Inhalation / exhalation) blood (or plasma) : air partition coefficient | Dimensionless | (Bessems et al., 2014; Chen et al., 2024) |
| ***Pharmacokinetics: General*** | | | | |
| ***AUC*** |  | Total area under the curve of chemical concentration in the systemic circulation as a function of time | Mass Volume ^-1^ Time ^-1^ | (Bessems et al., 2014; Buxton & Benet, 2011; EMA, 2018; Renwick, 2001) |
| ***C*_max_** |  | Maximum concentration in blood, plasma, or a tissue | Mass Volume^-1^ | (Bessems et al., 2014; EMA, 2018; OECD, 2021) |
| ***K*** |  | Rate constant | Time^-1^ | (OECD, 2021) |
| ***t*_1/2_** |  | Elimination half-life | Time | (EMA, 2018; OECD, 2021) |
| ***T*_max_** |  | Time to reach *C*_max_ | Time | (Bessems et al., 2014; OECD, 2021) |
| ***V*_d_** |  | Volume of distribution ^c^ | Volume | (Buxton & Benet, 2011; OECD, 2021) |
| ***Physiology*** | | | | |
| ***BW*** |  | Body weight | Mass | (Chen et al., 2024; Loccisano et al., 2011) |
| ***QC*** | *QCC; QC; Qc; CO* | Cardiac output | Volume Time^-1^ ; and variations  (e.g. L h^-1^ kg^-0.75^)^d^ | (Brown et al., 1997; Chen et al., 2024; Emond et al., 2010; Stader et al., 2019); (Loccisano et al., 2011) ^d^ |
| ***Q*_tissue_** (if given in absolute units such as mL min^-1^) ;  ***FQ*_tissue_** (if given as a fraction of the cardiac output) | *Qt; Q; QTC* (where *“T” was* replaced by the first letter/s of the tissue name, e.g. *QLC* for liver) | Blood flow to a tissue / organ | Volume Time^-1^ ;  Fraction of cardiac output | (Brown et al., 1997; Buxton & Benet, 2011; Chen et al., 2024; Emond et al., 2010; Loccisano et al., 2011) |
| ***W*_tissue_** (if given in absolute mass units) ;  ***FW*_tissue_** (if given as a fraction of BW) | *F*_tissue_*; VTC* (where *“T”* was replaced by the first letter/s of the tissue name, e.g. VLC for liver); *Vt0* | Weight ^f^ of a tissue /organ | Mass ; (Volume) ^f^ ;  Fraction of body weight | (Brown et al., 1997; Chen et al., 2024; Emond et al., 2010; Loccisano et al., 2011) |
| ***S*_skin_** | *Skin_area* | Total skin area | Area | (Chen et al., 2024) |
| ^a,b^ The subscript “tissue” may be replaced by the specific tissue or organ name (e.g. liver, fat…). The subscript “chemical" may be replaced by the abbreviation of the chemical substance. “Route” may be replaced by the specific exposure route (oral, dermal, inhalation). “Process"may be replaced by an abbreviation of the precise metabolic process.  ^a^ In PBPK models representing more than one chemical species, the abbreviation for the chemical substance may also be added to pharmacokinetic parameter abbreviations.  ^c^ for clarification, Vd is not a real body volume. It is the imaginary volume which would be needed to contain all the chemical in the body at the same concentration as in the systemic circulation (Buxton and Benet, 2011).  ^d^ In Loccisano et al. (2011), the units of Kurine and QCC (and Tmc, not shown) were not fully clear  ^e^ Attention, in Stader et al. (2019), Kapp was a constant for the apparent mechanism-based enzyme inhibition  ^f^ In PBPK models, weight (in reality, mass, kg, g) and volume (L, mL) are considered equivalent for most tissues. In fact in PBPK models, a mass-to-volume conversion may not be needed for the majority of organs, with density values close to 1 g/mL (1.02-1.06), although there are exceptions for certain tissues (Brown et al., 1997, p. 432). | | | | |

Table S2: steps for model verification and curation. The specific requirements for PBPK models are given in bold.

| Type of information | Recommendations and explanation | References |
| --- | --- | --- |
| Code verification | - Consistency between the mathematical model and the model code, - Complete information contained within the code, as explained in Table 7 | (ASME, 2018; FDA, 2023; Le Novere et al., 2005; Tiwari et al., 2021) |
| Model verifications conducted through simulation | - Calculation verification (see definitions in Table 1), - **Verification of the biological plausibility of the model (e.g. balance between the body weight and the weight of organs, between the cardiac output and the blood flows to organs).** - **Identification of the model uncertainties, such as possible identifiability issues during parameter estimation.** - **Sensitivity analysis for parameters.** - **Model validation (**see definitions in Table 1**).** | (Andersen et al., 1995; EMA, 2018; Le Novere et al., 2005; Loizou et al., 2008; Najjar et al., 2022; OECD, 2021; Tiwari et al., 2021) |

^1^ This refers to the peer-reviewed journal article or single reference description, where the mathematical model is described (Le Novere et al., 2005).

^2^ The model code or “computational model” is the implementation of the mathematical model in software (Le Novere et al., 2005; OECD, 2021; ASME V&V 40-2018, cited by FDA, 2023).

Table S3: important annotations (descriptive information) for the model developed in the current article. The annotations follow the guidelines provided in Table 5 in the manuscript.

| **Type of information** | **Correspondence in our model** |
| --- | --- |
| Model name | - PBPK_code_EDR_et_al_vs4 |
| Main files | - Current journal article (it includes the description of the mathematical model). - Original model code in MATLAB and SBML formats. Available in ZENODO (<https://doi.org/10.5281/zenodo.13838845>). |
| Journal article authors | - E. Domínguez-Romero, S. Mazurenko, M. Scheringer, V. Martins dos Santos, C. Evelo, M. Anton#, J. Hancock#, A. Županič#, M. Suarez-Diez. - # last authors (this implies major contributions) - Affiliations and funding sources shown in the current article. - ORCIDs:   - EDR: 0000-0001-9795-5628   - SM: 0000-0003-3659-4819   - MS: 0000-0002-0809-7826; <https://scholar.google.com/citations?user=_il-G0UAAAAJ&hl=en&oi=ao>   - VMDS: 0000-0002-2352-9017   - CE: 0000-0002-5301-3142   - MA: 0000-0002-7753-9042   - JH: 0000-0003-2991-2217   - AŽ: 0000-0003-3303-9086   - MSD: 0000-0001-5845-146X |
| Model code authors | - E. Domínguez-Romero##, S. Mazurenko## and M. Scheringer. - ## these authors contributed equally to writing the code |
| Code sources | - The MATLAB code proposed here is original. It has been written in MATLAB, from scratch, by the code authors. The SBML version was obtained by conversion of the MATLAB code, using MOCCASIN and COPASI (see Material and Methods). - No literature codes have been used. - No artificial intelligence tools have been used. |
| Model code ^3^ version | - Current: version 4 (September 9, 2024). - Preliminary versions of this code, written by the same authors: vs. 1a (August 7, 2023), vs. 1b (August 14, 2023), vs. 2 (May-June 6, 2024), vs. 3 (July 10, 2024). |
| Additional files | - Quantitative model simulation results: when the model code is executed, it creates an Excel file with quantitative model simulation results. This file is also available in Zenodo (<https://doi.org/10.5281/zenodo.12707615>). |
| License | - Journal article: license provided by the journal. - Model code, all versions: CC BY 4.0. - To cite a preliminary version of the code, please cite this journal article (Dominguez-Romero et al.), specify the code version used and explain any changes done by the users on the original version. |
| Naming of model components | The names of the model components, their values, units and sources are shown in Tables 2 and 3 in the manuscript. (The model equations are shown in Table 4). |

|  |  |
| --- | --- |

Figure S1: Simulated concentrations of chemical in blood (mg/L), liver and other tissues (mg/Kg), obtained by simulation of the proposed model code in MATLAB (left), and by execution of the SBML code in COPASI (right).

Simulation of the code by Dominguez-Romero et al., 2024, version 4. MATLAB R2019b and MATLAB free online (Basic) versions were used to write the code. The current simulation Figure was obtained by using MATLAB free online version (Basic). The conceptual PBPK model was adapted from a model by (Upton et al., 2016), as explained in the Material and Methods. As an example, the same exposure scenario as in (Upton et al., 2016) was used.

## Recommendations for naming and abbreviations of model components, notably parameters and state variables

To create a clear model code, the abbreviations used in the code should also be easy to follow. More generally, to make PBPK models more comprehensible, it is important to use the same abbreviation (and subscript) when referring to the same process. In PBPK modeling, some abbreviations are common and more or less harmonized by use, for example *BW* for the body weight and *Q* for blood flows. However for most processes and model components in PBPK models, the abbreviations and subscripts differ between models. In Table S1, we show some examples of abbreviations used in the literature for important processes and model components. For each process, we propose one or two abbreviations which seem the most clear and self-explanatory to us. This list is not exhaustive for all the abbreviations that have been used for the same process in different models, we just show some examples, or for all the processes that can be represented in PBPK models, we include major processes (Table S1).

As shown in Table S1, abbreviations are often used to specify model components and these may include subscripts.

- Physiological parameters may need subscripts to refer to one precise tissue or organ. We recommend using the full tissue names as subscripts. As an alternative, if tissue names are abbreviated, using abbreviations with at least 3 letters facilitates their understandability, such as “liv” for the liver or “thyr” for the thyroid. For the anatomical and physiological description of PBPK models, it may be useful to use terms to be found in a formal anatomy ontology, for example the Class “Anatomic Structure, System or Substance” (which includes and defines organs, tissues and fluids) in the “National Cancer Institute Thesaurus (NCIT)” ontology which can be found in BioPortal , (BioPortal, 2024; Whetzel et al., 2011). In principle, purely physiological parameters are independent of the chemical substance in PBPK models, since the potential impacts of chemical toxicity on physiology or anatomy are generally not studied in these models.
- Pharmacokinetic parameters result from interactions between physiology and chemicals. In Table S1, we propose abbreviations for important pharmacokinetic processes. Subscripts for tissues are important when one process (for example, partitioning) occurs in more than one tissue. Additionally, subscripts for the chemical substance may also be needed for models with more than one chemical species. For example, we propose to use *PC*_tissue_ or *PC*_tissue_blood_ for the partition coefficient of a chemical between a tissue and the blood. If a model includes more than one chemical species, we could complete these abbreviations with the subscript for the chemical, as follows: *PC*_tissue_chemical_ or *PC*_tissue_blood_chemical_, respectively. As an example, the PC for diethyl phthalate (DEP) in liver could be abbreviated as: *PC*_liver_DEP_ or *PC*_liver_blood_DEP_. Chemical abbreviations may be found in the European Chemicals Agency (ECHA) website (ECHA, 2024), on PubChem (NIH, 2024), and others (see Madden et al. (Madden et al., 2019)). Importantly, some scientific publications and reviews for complex chemical families include lists of abbreviations generally used in the literature for those substances (e.g. Domínguez-Romero et al. (Domínguez-Romero et al., 2023)).
- Physico-chemical parameters, not necessarily specified in PBPK models and not included in Table S1, are independent of physiology. If a model is developed for more than one chemical, these parameters may require subscripts for the chemical substance of interest. For example, the logarithm of the octanol-water partitioning (log *K*_ow_) of DEP could be abbreviated as *logKow*__DEP_.

## Code re-use: examples of potential modifications on the code

The proposed code simulates the mathematical model we explain in the manuscript. Nevertheless, the code is flexible and can be adapted to represent other models. For example, new compartments can be added, by duplicating and adapting the general equation of others (other tissues). This will create new parameters for that compartment (blood flow, tissue weight, partition coefficient), which should be characterized (in terms of their values, units and sources). As explained within the code, if a new tissue / compartment (currently contained within others) is explicitly added to the model, the weight and blood flow from that tissue / organ should then be extracted from those of others, to maintain the biological plausibility of the model. Concerning exposure routes, to represent for example oral exposure in our model, it is possible to add a gut compartment linked to the liver through the portal vein flow (Loccisano et al., 2011), with the possibility to add an absorption coefficient if needed (Teeguarden et al., 2005). If oral exposure, the gut compartment, and the portal vein blood flow were explicitly added, the portal vein blood flow should be subtracted from the current total flow to the liver. Other pharmacokinetic processes such as binding to proteins could be added and represented in a simple manner, as in Loccisano et al. (Loccisano et al., 2011). To represent the pharmacokinetics of more than one chemical substance (e.g. one parent chemical and one or more metabolites), a *mirror* submodel can be added (Chen et al., 2024)).

In the end, it is important to ensure that all new components are abbreviated, defined and parameterized clearly and consistently in all model files, and to evaluate and validate the resulting model for its purpose.

## References

Andersen, M. E., Clewell, H. J., & Frederick, C. B. (1995). Applying Simulation Modeling to Problems in Toxicology and Risk Assessment: A Short Perspective. *Toxicology and Applied Pharmacology*, *133*(2), 181-187. <https://doi.org/https://doi.org/10.1006/taap.1995.1140>

ASME. (2018). *Assessing Credibility of Computational Modeling through Verification and Validation: Application to Medical Devices,   V 40 - 2018 (cited by FDA, 2023)* (ASME, Ed.). ASME.

Bessems, J. G., Loizou, G., Krishnan, K., Clewell, H. J., Bernasconi, C., Bois, F.,…Zaldivar-Comenges, J. M. (2014). PBTK modelling platforms and parameter estimation tools to enable animal-free risk assessment Recommendations from a joint EPAA - EURL ECVAM ADME workshop. *Regulatory Toxicology and Pharmacology*, *68*(1), 119-139. <https://doi.org/10.1016/j.yrtph.2013.11.008>

BioPortal. (2024). *National Cancer Institute Thesaurus Last uploaded: February 23, 2024*. Retrieved September 13, 2024 from <https://bioportal.bioontology.org/ontologies/NCIT/?p=summary>

Brown, R. P., Delp, M. D., Lindstedt, S. L., Rhomberg, L. R., & Beliles, R. P. (1997). Physiological parameter values for physiologically based pharmacokinetic models. *Toxicology and Industrial Health*, *13*(4), 407-484. <https://doi.org/https://doi.org/10.1177/074823379701300401>

Buxton, I. L. O., & Benet, L. Z. (2011). *Pharmacokinetics: the Dynamics of Drug Absorption, Distribution, Metabolism, and Elimination. Goodman & Gilman's the Pharmacological Basis of Therapeutics. 12th ed.* McGraw-Hill, pp. 17-40.

Chen, S., Shi, Z., & Zhang, Q. (2024). A physiologically based pharmacokinetic model of diethyl phthalates in humans. *Environmental Pollution*, *340*, 122849. <https://doi.org/https://doi.org/10.1016/j.envpol.2023.122849>

Domínguez-Romero, E., Komprdová, K., Kalina, J., Bessems, J., Karakitsios, S., Sarigiannis, D. A., & Scheringer, M. (2023). Time-trends in human urinary concentrations of phthalates and substitutes DEHT and DINCH in Asian and North American countries (2009-2019). *Journal of Exposure Science and Environmental Epidemiology (*[*https://www.nature.com/jes/*](https://www.nature.com/jes/)*)*, *33*(2), 244-254. <https://doi.org/10.1038/s41370-022-00441-w>; <https://www.nature.com/articles/s41370-022-00441-w>

ECHA. (2024). *European Chemicals Agency (ECHA). Search for chemicals (tool).* Retrieved April 13, 2024 from <https://echa.europa.eu/information-on-chemicals>

EMA. (2018). *Guideline on the reporting of physiologically based pharmacokinetic (PBPK) modelling and simulation.  European Medicines Agency (EMA), Committee for Medicinal Products for Human Use (CHMP).  13 December 2018. 16 pp. EMA/CHMP/458101/2016.*

Emond, C., Raymer, J. H., Studabaker, W. B., Garner, C. E., & Birnbaum, L. S. (2010). A physiologically based pharmacokinetic model for developmental exposure to BDE-47 in rats. *Toxicology and Applied Pharmacology*, *242*(3), 290-298. <https://doi.org/10.1016/j.taap.2009.10.019>

FDA. (2023). *Assessing the Credibility of Computational Modeling and Simulation in Medical Device Submissions. Guidance for Industry and Food and Drug Administration Staff. Document issued on November 17, 2023. 42 pp. U.S. Department of Health and Human Services. Food and Drug Administration Center for Devices and Radiological Health.*

Le Novere, N., Finney, A., Hucka, M., Bhalla, U. S., Campagne, F., Collado-Vides, J.,…Wanner, B. L. (2005). Minimum information requested in the annotation of biochemical models (MIRIAM). *Nature Biotechnology*, *23*(12), 1509-1515. <https://doi.org/10.1038/nbt1156>

Loccisano, A. E., Campbell, J. L., Andersen, M. E., & Clewell, H. J. (2011). Evaluation and prediction of pharmacokinetics of PFOA and PFOS in the monkey and human using a PBPK model. *Regulatory Toxicology and Pharmacology*, *59*(1), 157-175. <https://doi.org/10.1016/j.yrtph.2010.12.004>

Loizou, G., Spendiff, M., Barton, H. A., Bessems, J., Bois, F. Y., d'Yvoire, M. B.,…Schmitt, W. (2008). Development of good modelling practice for physiologically based pharmacokinetic models for use in risk assessment: The first steps. *Regulatory Toxicology and Pharmacology*, *50*(3), 400-411. <https://doi.org/10.1016/j.yrtph.2008.01.011>

Madden, J. C., Pawar, G., Cronin, M. T. D., Webb, S., Tan, Y. M., & Paini, A. (2019). *In silico* resources to assist in the development and evaluation of  physiologically-based kinetic models. *Computational Toxicology*, *11*, 33–49. <https://doi.org/https://doi.org/10.1016/j.comtox.2019.03.001>

Najjar, A., Punt, A., Wambaugh, J., Paini, A., Ellison, C., Fragki, S.,…Kramer, N. I. (2022). Towards best use and regulatory acceptance of generic physiologically based kinetic (PBK) models for in vitro-to-in vivo extrapolation (IVIVE) in chemical risk assessment. *Archives of Toxicology*, *96*(12), 3407-3419. <https://doi.org/10.1007/s00204-022-03356-5>

NIH. (2024). *PubChem. Explore Chemistry. National Library of Medicine, National Center for Biotechnology Information*. <https://pubchem.ncbi.nlm.nih.gov/>

OECD. (2021). *Guidance document on the characterisation, validation and reporting of Physiologically Based Kinetic (PBK) models for regulatory purposes, OECD Series on Testing and Assessment, No. 331,  101 pp. Environment, Health and Safety, Environment Directorate, OECD.*

Renwick, A. G. (2001). Toxicokinetics: pharmacokinetics in toxicology. Principles and Methods of Toxicology, fourth edition. In A. W. Hayes (Ed.), (pp. 137-191). Taylor & Francis.

Stader, F., Penny, M. A., Siccardi, M., & Marzolini, C. (2019). A Comprehensive Framework for Physiologically-Based Pharmacokinetic Modeling in Matlab. *Cpt-Pharmacometrics & Systems Pharmacology*, *8*(7), 444-459. <https://doi.org/10.1002/psp4.12399>

Teeguarden, J. G., Waechter, J. M., Clewell, H. J., Covington, T. R., & Barton, H. A. (2005). Evaluation of oral and intravenous route pharmacokinetics, plasma protein binding, and uterine tissue dose metrics of bisphenol A: A physiologically based pharmacokinetic approach. *Toxicological Sciences*, *85*(2), 823-838. <https://doi.org/10.1093/toxsci/kfi135>

Tiwari, K., Kananathan, S., Roberts, M. G., Meyer, J. P., Sharif Shohan, M. U., Xavier, A.,…Malik-Sheriff, R. S. (2021). Reproducibility in systems biology modelling. *Molecular systems biology*, *17*(2), e9982. <https://doi.org/10.15252/msb.20209982>

Upton, R. N., Foster, D. J. R., & Abuhelwa, A. Y. (2016). An introduction to physiologically-based pharmacokinetic models. *Pediatric Anesthesia*, *26*(11), 1036-1046. <https://doi.org/10.1111/pan.12995>

Whetzel, P. L., Noy, N. F., Shah, N. H., Alexander, P. R., Nyulas, C., Tudorache, T., & Musen, M. A. (2011). BioPortal: enhanced functionality via new Web services from the National Center for Biomedical Ontology to access and use ontologies in software applications. *Nucleic Acids Res*, *39*(Web Server issue), W541-545. <https://doi.org/10.1093/nar/gkr469>
